# The Impact of Diet on AD Biomarkers in a Community‐Based Sample

**DOI:** 10.1002/alz.093624

**Published:** 2025-01-09

**Authors:** Amarachukwu Okafor, Ambika Vartak, Aubrey S. Johnson, Diana S. Guzmán, Anna C. Smith, Hannah M. Houlihan, Lauren B. Heuer, Miguel Arce Rentería, Indira C. Turney, Richard Mayeux, Adam M. Brickman, Jennifer J. Manly, Yian Gu, Patrick J. Lao

**Affiliations:** ^1^ Columbia University Irving Medical Center, New York, NY USA; ^2^ Mailman School of Public Health, New York, NY USA; ^3^ Department of Neurology, Columbia University, New York, NY USA

## Abstract

**Background:**

Greater adherence to the Mediterranean Diet (MeDi) is associated with lower risk for cardiovascular disease, slower cognitive decline, and reduced risk for Alzheimer’s Disease (AD). However, its association with AD biomarkers is not well known. We hypothesized that greater MeDi adherence is associated with reduced amyloid and tau PET burden in a community‐based sample of older adults in Northern Manhattan.

**Methods:**

Participants from the Washington Heights/Inwood Columbia Aging Project (WHICAP) completed the Willet Food Frequency questionnaire (FFQ) at study baseline. A subset completed one (n=161 participants; age=81±6 years, 20% Hispanic, 50% Black, 65% women, education=13±4 years) or two timepoints of amyloid PET (FBB, n=19; follow‐up time=6.4±1.9), as well as one timepoint of tau PET (MK‐6240, n=17). PET scans were 8.6±4.6 years after the FFQ. We evaluated the impact of MeDi adherence on subsequent baseline amyloid and tau burden, quantified as SUVR or positivity (SUVR>1.25 for amyloid or SUVR>1.81 for tau), as well as the annualized percent change in amyloid burden over time, accounting for age, sex, race and ethnicity, education, caloric intake, and time between the FFQ and PET scan.

**Results:**

Fifty‐eight (36%) participants were amyloid positive and 7 (41%) were tau positive in early Braak regions at their first timepoint, and 2 (8%) individuals converted from amyloid negative to amyloid positive at their second timepoint. Greater MeDi adherence was not associated with amyloid burden (‐0.02 [‐0.04, 0.004], p=0.11) but was marginally associated with a lower likelihood of amyloid positivity (‐0.21 [‐0.44, 0.0009], p=0.055). Greater MeDi adherence was not associated with tau burden (0.003 [‐0.13, 0.13], p=0.97) or the likelihood of tau positivity (‐0.66 [‐1.9, 0.63], p=0.3). MeDi adherence was not associated with the change in amyloid burden over time (‐0.19 [‐1.5, 1.1], p=0.8).

**Conclusion:**

Greater MeDi adherence was associated with a reduced likelihood of subsequent amyloid positivity, suggesting diet may play a role in amyloid‐related AD risk. Replication in larger studies is needed. Further attention should be placed on the individual components of the MeDi adherence score (e.g. fish, vegetables) and their differential consumption and effects across racial and ethnic groups.

